# The Spring

**Published:** 1841-06

**Authors:** 


### THE SPRING.

Till within the last fortnight, which has brought us Summer heat and drought, the Spring has been cold, with an extraordinary prevalence of East and Northeast winds; and the Ohio has kept at an elevation indicative of much rain within its borders. Vegetation has of course been backward. We hope that our brethren have attentively marked the effects of this kind of weather on our vernal diseases; and that some of them will in due time give us the results of their observation. D.

---

We learn that Dr. Bartlett, of Lowell, Mass., has been appointed to the chair of Theory and Practice in Transylvania University.

